# Functional materials for environmental sensors and energy systems

**DOI:** 10.3762/bjnano.8.201

**Published:** 2017-09-26

**Authors:** Michele Penza, Anita Lloyd Spetz, Albert Romano-Rodriguez, Meyya Meyyappan

**Affiliations:** 1 ENEA - Laboratory Functional Materials and Technologies for Sustainable Applications, Brindisi Research Center, P.O. Box 51 Br4, 72100 Brindisi, Italy; 2 Department of Physics, Chemistry & Biology, Linköping University, SE-581 83 Linkoping, Sweden; 3 Department of Electronics, University of Barcelona, 08028 Barcelona, Spain,; 4 Ames Research Center, NASA, 94035 Moffett Field, CA, USA

**Keywords:** air-quality monitoring, chemical sensors, energy systems, functional materials, sensor materials

Advanced materials and functional nanomaterials continue to intrigue researchers and scientists, providing a source of continuous discovery from basic to applied research. This field of research has been useful for open innovation with the general purpose of achieving a better, more sustainable world with cleaner air.

Functional nanomaterials (e.g., nanowires, nanotubes, graphene, metal oxides, carbon nanostructures, large band gap semiconductors, and metals) with new sensing properties (e.g., ppb-level detection, high sensitivity, selectivity) that are self-heating and provide durable operation for low-power devices (tens of μW to tens of mW) are key elements for adequate air-quality measurement at indoor and outdoor levels.

This Thematic Series, published in the platinum open access *Beilstein Journal of Nanotechnology*, is an exciting collection of works on functional nanomaterials such as metal oxides, carbon nanomaterials (e.g., nanotubes, graphene-based materials), 2D nanostructures, hybrid materials and their related surface modifications in order to enhance the sensing properties. Particular emphasis was placed on rapidly emerging applications in the sector of air-quality monitoring and energy systems for environmental sustainability. The participation of a substantial number of prominent authors from COST Action TD1105 EuNetAir [1] (European Network on New Sensing Technologies for Air-Pollution Control and Environmental Sustainability) made this a highly successful endeavor. Inspiration for this Thematic Series was drawn from original research presented as invited, oral and poster presentations at the 5-day Symposium X [2] at the European Materials Research Society (E-MRS) Spring Meeting 2016, May 2–6, in Lille, France, which was devoted to advanced functional materials for chemical sensors and energy systems.

Nanotechnology faces a big challenge to create innovative, low-cost sensors for air-quality monitoring and energy systems applications. The development of functional nanomaterials with new, tailored properties is a key issue for the improvement of low-power devices for indoor and outdoor air-quality monitoring. This includes practical applications such as geo-tagged databases that are collected by networked, stationary or mobile smart devices to address new sensing concepts for air-quality monitoring as well as mapping techniques of gas molecules and particulate matter [3–5]. These solid-state chemical sensors based on smart materials with autonomous operation and low-power consumption are useful for real deployment and are complementary to the existing, high-cost, high-accuracy air-quality monitoring stations used by public authorities. These new, cost-effective sensor systems will be beneficial for the scientific community, policy makers and social networks.

The topics of this Thematic Series, based on 23 peer-reviewed articles, includes works on advanced gas sensing semiconducting materials, hybrid materials and nanocomposites for chemical sensing, catalytic sensing materials, metal oxides for chemical sensing and energy applications, carbon-based materials for chemical sensing and energy applications, piezoelectric and thermoelectric materials for energy harvesting applications, new nanotechnology-based sensors for monitoring gaseous and liquid pollutants, surface-sensitive spectroscopy for studying sensor/gas interaction, modelling of materials, devices, sensor systems and energy systems, and functional applications of environmental sensors and energy systems.

Michele Penza, Anita Lloyd Spetz, Albert Romano-Rodriguez and Meyya Meyyappan

Brindisi, Linkoping, Barcelona and Moffett Field, May 2017
